# 

**Published:** 1915-06

**Authors:** 


### ANNOUNCEMENTS.

THE PANAMA-PACIFIC DENTAL CONGRESS is to be held at San Francisco, California, in the Exposition Auditorium, August 30, to September 9, 1915.

The members of the Transportation Committee of the National Dental Association, adopted three official railway routes and schedules to San Francisco from the East, and are now engaged in calling the attention of the profession to the schedules planned. Members of the committee are located in different sections of the states, and are prepared to give members of the profession in their localities, general information regarding the railway routes, fares, etc.

The railway trains scheduled as arranged by the committee going to San Francisco have special equipment, and train service. The routes are popular, the trains are of the most comfortable cars; the arrangements being complete, insures pleasant associations and encourages good fellowship.

The Railway Itinerary of the official trains as published in the February number of the *Dental Register,* page 94, should be examined carefully by all that are intending to make the trip, as the plan adopted in employing our special and official trains is to encourage comfort in transit and general good fellowship. The committee suggests that one should confer with their local railway agent, or one referred to in our itineraries, and choose a route for their return trip, which is necessary before purchasing a reduced fare ticket.

The committee request that editors of all dental journals, officers of state and local dental societies and all members of the profession do what they can to interest members of the profession in joining us in our trip to attend the Congress, and in that manner add to the attendance and assure the success of the Congress.

Transportation Committee National Dental Association: Dr. Victor H. Jackson (Chairman), 40 E. 41st St., N. Y.; Dr. H. F. Hoffman, 324 Metropolitan Bldg., Denver, Colo.; Dr. Jos. D. Eby, 509 Fourth Nat. Bk. Bldg., Atlanta, Ga.; Dr. D. C. Bacon, Columbus Memorial Bldg., Chicago, Ill.; Dr. Henry W. Weirick, 503 Mechanics Bldg., San Francisco, Cal.; Dr. J. P. Marshall, 7401 Hazel Ave., St. Louis Mo.

---

F. D. I.—The next meeting of the International Dental Federation will be held September 2-3, 1915, at San Francisco, California. Headquarters will be at The Cliff Hotel. Truman W. Brophy, President; Burton Lee Thorpe, Assistant Secretary, 3605 Lindell Blvd., St. Louis, Mo.

---

PANAMA-PACIFIC DENTAL CONGRESS.—The Committee of Organization of the Panama-Pacific Dental Congress desires to call the attention of the members of the dental profession to the fact that the Congress will convene on time in San Francisco, on August 30th, under most favorable conditions for holding a large and successful meeting.

The program of papers and clinics is almost complete and covers in a most comprehensive manner practically every subject pertaining to the practice of dentistry and oral surgery. About one hundred papers and two hundred and fifty clinics, or more, will be presented. Among the leading essayists and clinicians will be, Truman W. Brophy, H. S. Dunning, M. H. Cryer, C. H. Oakman, Rudolph Weiser, of Vienna, Austria, A. B. Baer, T. B. Hartzell, H. M. Sherman, T. E. Carmody, W. H. G. Logan, H. P. Carlton, Prof. Bornsdorff, of Finland, E. F. Leffler, Garrett Newkirk, Herbert L. Wheeler, Louis Ottofy, Guy S. Millberry, M. L. Ward, I. N. Broomell, H. E. Friesell, V. A. Latham, F. B. Noyes, Josef Novitsky, C. H. Wilson, F. W. Hergert, C. J. R. Engstrom, V. E. Mitchell, Hart J. Goslee, J. Leon Williams, Alfred P. Rogers, A. H. Ketcham, E. L. Stanton, Weston A. Price,

Luis Subirana, Madrid, Spain, R. Ottolengui, M. L. Rhein, H. A. Pullen, Vincenzo Guerini, of Italy, Joseph Nalin, of Montreal, W. H. Fitzgerald, Chas. McManus, C. O. Simpson, M. J. Congdon, R. B. Giffen, H. G. Chappel, Th. Weber, of Finland, A. C. Wherry, John V. Conzett, F. W. Gethro, Edwin R. Kibler, Richard H. Riethmuller, T. Sydney Smith, Robin Adair, H. Page Bailey, Wm. A. Capon, L. P. Haskell, Jules J. Serrazin, V. H. Jackson, W. H. O. McGehee, Arthur C. Peck, E. A. Bogue, and many others.

As far as possible no night sessions of the Congress will be held, leaving the evenings free for entertainment and the Exposition.

Over 1200 front feet of space will be occupied by the leading dealers and manufacturers of the world with one of the most comprehensive exhibits of dental and pharmaceutical goods ever shown.

All the sessions of the Congress, the meetings of the sections, component societies and the exhibits will be held under one roof, in the Municipal Auditorium, one of the most magnificent structures of its kind, affording every opportunity for the effective and comfortable presentation of the program.

The Committee of Organization is sparing neither time, labor or money, to make the Congress the most notable event if its kind in the history of dentistry. To have missed it will be the regret of a lifetime. Transportation and hotel accommodations will be within the reach of all, and should be secured at once. Over 1000 applications for membership are now on file with the Committee. Those who have not filed their application should do so now, so that arrangements may be made for their accommodation and entertainment.

No one interested in the history, progress and practice of dental science can afford to miss this great opportunity to attend the Congress and at the same time visit the greatest International Exposition the world has ever seen.

